# Effectiveness and Toxicity Profile of Reduced Dose Sorafenib for the Treatment of Hepatocellular Carcinoma: A Retrospective, Single Institutional Experience

**DOI:** 10.7759/cureus.73729

**Published:** 2024-11-15

**Authors:** Abhilash Menon, Nandini Devi R, Praveen K Shenoy, Manuprasad A Avaronnan, Allwin George

**Affiliations:** 1 Department of Clinical Hematology and Medical Oncology, Malabar Cancer Centre, Thalassery, IND; 2 Department of Medical Oncology, Aster - Malabar Institute of Medical Sciences (MIMS), Kannur, IND; 3 Department of Medical Oncology, Ayillyath Kuttiari Gopalan (AKG) Memorial Cooperative Hospital, Kannur, IND

**Keywords:** effective, hepatocellular carcinoma, low dose sorafenib, minimal effective dose, reduced toxicity

## Abstract

Background

The treatment with sorafenib in hepatocellular carcinoma (HCC) is affected by toxicity and discontinuation rates. There is limited data on whether ensuring compliance by reducing the dose of sorafenib can influence outcomes.

Methods

In this retrospective study, we used hospital records to retrieve data on patients treated with low-dose sorafenib (400 mg /day) from July 2017 to June 2022 at the Malabar Cancer Centre, Thalassery.

Results

During the study period, 80 patients received low-dose sorafenib for HCC. Sixty-eight (85%) patients were males with median age being 62 years, ranging from 17 to 79 years. More than three-fourths (76.2%) of the patients had Barcelona stage C and nearly one-third (31.2%) had Child-Pugh B status. Alcohol consumption and obesity were seen in 36 (45%) and 24 (30%) patients respectively. Clinical benefit rate (at least stable disease) at three months was seen for 45 (56.25%) patients. The median follow-up was six months. The median progression-free survival (PFS) and overall survival (OS) were 3.68 (CI 2.89-4.46) and 5.26 (CI 3.26-7.27) months respectively. Nine patients (11.25%) had grade 3 toxicity, and six (7.5%) patients stopped sorafenib due to toxicity despite dose reduction.

Conclusion

In comparison to other published landmark studies, our study demonstrates that reduced dose sorafenib in advanced hepatocellular carcinoma has a similar response rate and progression-free survival with lesser toxicity. In the real world, a reduced dose of sorafenib is nevertheless effective when tolerance and cost are concerns. Additionally, since a third of the study cohort has Child-Pugh B, a reduced dose of sorafenib may be a choice for these patients.

## Introduction

Hepatocellular carcinoma (HCC) is the eighth most common cancer among males in India [1]. Sorafenib is approved for use as first-line systemic chemotherapy in patients with incurable HCC. This is supported by two randomized phase III studies, the Asian Pacific study and the Sorafenib Hepatocellular Carcinoma Assessment Randomised Protocol (SHARP) study, which demonstrated that sorafenib therapy improved survival compared to the placebo group [2,3]. Other first-line options include atezolizumab plus bevacizumab, lenvatinib, and tremelimumab plus durvalumab. Most of the patients in our setting cannot afford immunotherapy. Lenvatinib is also not affordable for many and has tolerance issues. Hence sorafenib still plays a major role in the management of these patients

Sorafenib's recommended dose is 400 mg twice daily [2]. However, a large fraction of patients discontinue treatment because of adverse events including hand-foot skin reaction, rash, liver dysfunction, fatigue, and diarrhoea. The rate of serious side effects (AEs) in a phase 4 non-interventional study was 85.3%; 31.7% of patients experienced a grade 3 or 4 AE, and 31.4% of patients stopped taking sorafenib permanently as a result of an AE [4]. Controlling adverse events (AEs) through dose reduction can enhance drug adherence and treatment response [5]. The role of ethnicity in personalized dosing of tyrosine kinase inhibitors is often overlooked. Asians experience more hand-foot-skin reactions due to sorafenib [6]. The concept of the maximum tolerable dose is slowly being replaced by a biologically effective dose, especially for targeted agents [7].

An alternate dose-escalation approach was studied in a previous trial, wherein a subgroup of patients began on a 50% dose of sorafenib and was only increased if tolerability was maintained. The experimental group saw a lower incidence of adverse events and medication discontinuation [8]. In our institute, the Malabar Cancer Centre, Thalassery, we initiate sorafenib at half the dose of 400 mg once daily and consider escalation for those with good tolerance. The data on the efficacy and safety of reduced dose sorafenib in HCC is scarce, hence this study was done.

## Materials and methods

This was a retrospective observational study among patients with metastatic or local treatment non-amenable HCC patients treated with low-dose sorafenib (400 mg/day) from July 2017 to June 2022 at the Malabar Cancer Centre, Thalassery. The unique hospital identification number of patients on sorafenib was obtained from the pharmacy. The case records were then retrieved for collecting further details. The Institutional Review Board, Malabar Cancer Centre, approved the study (1616/IRB-SRC/13/MCC/23-09-2023/2). A waiver of consent was obtained due to the retrospective nature of the study. The study's primary objective was to determine the clinical benefit rate, progression-free survival (PFS), and toxicities of low-dose sorafenib. The secondary objective was to estimate the overall survival (OS) with low-dose sorafenib in HCC. Patients with incomplete data were excluded.

The following operational definitions were used. Clinical benefit rate was the percentage of patients with complete response, partial response, and stable disease as a result of the treatment. OS was calculated from the date of initiation of sorafenib treatment until death from any cause or the last follow-up. PFC was calculated from the initial date of sorafenib treatment until the date of progression of disease or death from any cause. Complete response (CR) was the complete disappearance of all lesions and lymph nodes. Partial response (PR) was at least a 30% decrease in the sum of the longest diameter of target lesions, taking as reference the baseline sum of the longest diameter. Progression was more than a 20% increase in the sum of the longest diameter of target lesions, taking as reference the baseline sum of the longest diameter or any new lesion. Stable disease was the response that did not fall into CR, PR, or progression.

All the patients were started on sorafenib at a dose of 400 mg once daily. They underwent their first clinical, haematological, and biochemical assessment at two weeks for tolerance, followed by a monthly visit. Toxicity grading was done using the Common Terminology Criteria for Adverse Events (CTCAE), version 5. The sorafenib dose was not escalated beyond the starting dose. For patients who experienced grade 3 or persistent grade 2 toxicity, the drug will be interrupted and restarted once it resolves at a lower dose level. The first radiological assessment was usually done along with serum AFP (alpha-fetoprotein) estimation three months after initiation of sorafenib. Further radiological assessments were as clinically indicated. Sorafenib was continued till progression or till grade 3/4 toxicity.

Statistics

Being a retrospective study, sample size calculation was not performed for this study. Patient characteristics were summarized with mean and standard deviation for continuous data, and with frequencies and percentages for categorical data. The Chi-square or Fischer’s exact test was used for the comparison of categorical variables. OS and PFS were estimated using the Kaplan-Meier method. Statistical analysis was done using the Statistical Package for the Social Sciences (SPSS), version 24 (IBM Corp., Armonk, New York, United States).

## Results

A total of 80 patients received low-dose sorafenib during the study period. The median age was 62 years (17-79 years). The baseline Eastern Cooperative Oncology Group (ECOG) performance status was PS-1 in 48 (60%) patients, and PS-2 in 32 (40 %). More than half of the patients (52.5%) had diabetes as a comorbidity. Seven patients were positive for Hepatitis B and four for Hepatitis C. Forty-five per cent of patients were alcoholic. The most common Barcelona stage was stage C (76.2%). Fifty-five patients (68.8%) had Child-Pugh A status while the rest were Child-Pugh B. Distant metastases were present in 23 patients. The most common site of metastases was bone followed by the lung. Seven patients underwent local treatment previously as their initial treatment. Baseline characteristics are shown in Table 1.

**Table 1:** Baseline characteristics BMI: Body Mass Index, SD: standard deviation, AST: aspartate aminotransferase, ALT: alanine aminotransferase, AFP: alfa-fetoprotein, TACE: trans-arterial chemoembolization, MWA: microwave ablation, RFA: radiofrequency ablation, ECOG: Eastern Cooperative Oncology Group

Variable	Value
Median age in years (range)	62 (17-79)
Males-n (%)	68 (85%)
Females – n (%)	12 (15%)
Mean BMI (SD)	23.28 (4.07)
Comorbidities	
Diabetes	42 (52.5%)
Hypertension	28 (35%)
Aetiology	
Alcoholic	36 (45%)
Hepatitis B	7 (8.8%)
Hepatitis C	4 (5.5%)
Obesity (BMI >=25.0 kg/m²)	24 (30%)
Biochemical parameters	
Median bilirubin	1.3 (0.1-19.2)
Mean albumin (SD)	3.58 (0.55)
Median AST (range)	85 (32-518)
Median ALT (range)	52 (15-196)
Median AFP (range)	753 (19-357330)
Barcelona Stage	
Stage B	19 (23.7%)
Stage C	61 (76.2%)
Child-Pugh status	
Child-Pugh A	55 (68.8%)
Child-Pugh B	25 (31.2%)
Extrahepatic spread	
Regional lymph node	10 (12.5%)
Metastases	23 (28.8%)
Prior procedure	
Surgery	1 (1.3%)
TACE	3 (3.8%)
RFA	2 (2.5%)
MWA	1 (1.3%)
ECOG performance status	
1	48 (60%)
2	32 (40%)

Outcome

**Figure 1:**
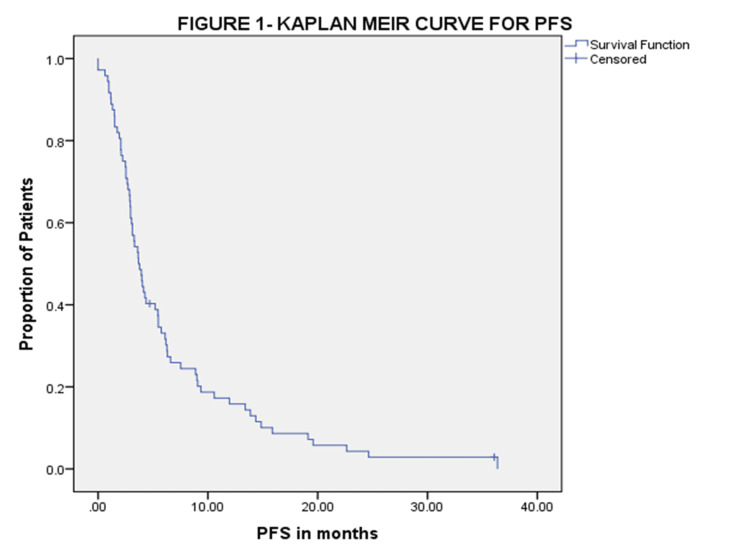
Kaplan-Meier curve showing PFS PFS: progression-free survival

**Figure 2:**
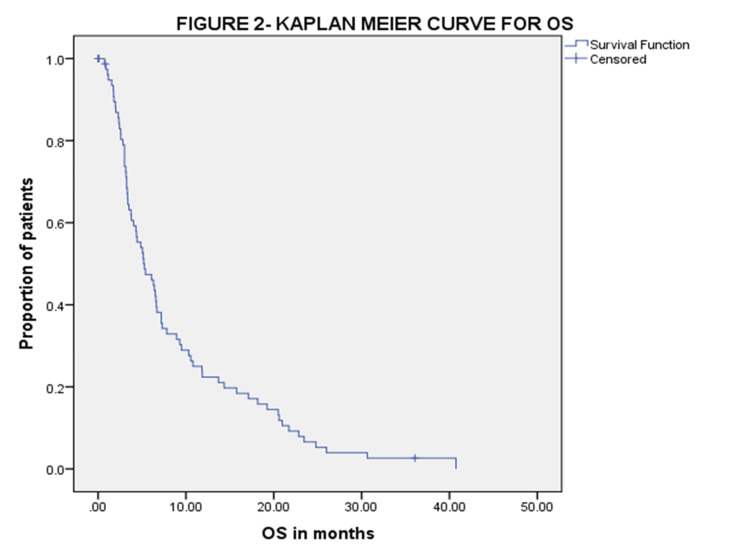
Kaplan-Meir curve showing OS OS: overall survival

At three months of assessment, six (7.5%) patients had a partial response, 39 (48.75%) patients had stable disease, 25 (31.25%) patients progressed, six (7.5%) patients stopped the drug due to poor tolerance, and four (5%) defaulted. Clinical benefit at three months was seen for 45 (56.25%) patients. The median follow-up was six months. The median PFS and OS were 3.68 (95% CI 2.89-4.46) and 5.26 (95% CI 3.26-7.27) months. The median PFS was 3.94 months (95% CI 3.08-4.79) in Child-Pugh A and 3.1 months (95% CI 2.40-3.83) in Child-Pugh B with a hazard ratio of 0.932 (95% CI 0.517-1.68, p-0.76). The median OS was 6.4 months (95% CI 4.7-8.1) in Child-Pugh A and 4.3 months (95% CI 2.3-6.2) in Child-Pugh B with a hazard ratio of 1.03 (95% CI 0.62-1.7 p-0.85). The Kaplan-Meier curves for PFS and OS are shown in Figure 1 and Figure 2 respectively.

Toxicities 

Eleven patients had treatment interruptions due to toxicities, and six patients stopped sorafenib due to toxicity despite dose reduction. Table 2 shows the grade 2 and grade 3 toxicities experienced by the patients. The most common grade 2 and grade 3 toxicity observed was fatigue.

**Table 2:** Treatment-emergent adverse events

Toxicity	Grade 2	Grade 3
Fatigue	21 (26.2%)	5 (6.25%)
Hand-foot-skin reaction	13 (16.25%)	-
Mucositis	7 (8.75%)	-
Skin rash	6 (7.5%)	3 (3.75%)
Diarrhoea	4 (5%)	1 (1.25%)

## Discussion

The study showed that sorafenib at a reduced dose of 400 mg daily resulted in a comparable response rate and progression-free survival similar to other landmark studies with fewer adverse events. The median age of our study is comparable with other studies. More than three-fourths of our patients had Barcelona stage C. Hepatitis B or C positivity was seen in around 14%, which is very low compared to other reported studies, including those from Asia. Forty-five per cent of patients were alcoholics. This reflects the difference in the predominant aetiology of HCC in the Indian population compared to other parts of the world. A recent 2024 update on the epidemiology of HCC in India by Giri and Singh suggests the changing etiological pattern of cirrhosis and HCC, with alcohol and metabolic dysfunction-associated steatotic liver disease (MASLD) emerging as the foremost cause [9]

The clinical benefit rate defined by the percentage of patients with complete response, partial response, and stable disease was 56% almost similar to other studies [2,3]. Median PFS was 3.68 months which is comparable to other reported studies. This is notable because nearly one-third of our patients had Child-Pugh B status. OS in our study was comparatively less compared to other studies [2,3]. In our population access to second-line therapies was limited because of financial constraints and this may account for poor OS among our patients. Around 30% of patients in our study had Child-Pugh B status at the start of sorafenib. It is likely they might have experienced deterioration at progression. This could be another explanation for poor OS among our patients. In the REFLECT (A Multicenter, Randomized, Open-Label, Phase 3 Trial to Compare the EFficacy and Safety of LEnvatinib (E7080) Versus Sorafenib in First-Line Treatment of Subjects With UnreseCtable HepaTocellular Carcinoma) study, 39% of patients received subsequent treatment while in our study only five patients received subsequent treatment [10]. In the real-world setting, a retrospective study from Veterans Health Administration hospitals showed a median survival of 200 days with a reduced dose of sorafenib which is similar to our study [11]. The most common toxicity in our study was fatigue, which is different from the SHARP, REFLECT, and IMbrave150 study, where it was diarrhoea and hand-foot syndrome (HFS) in the Asia-Pacific study [2,3,10,12]. The incidence of HFS was also low, this may be explained by the reduced dose at which sorafenib was given. A study by Ostwal V et al. with full dose sorafenib showed that the discontinuation rate was 41%, much higher than our study (7.5%) [13]. Ethnicity does have a role in efficacy and toxicity as observed in our study where the pattern of toxicities was different compared to other studies. Table 3 compares our study with other similar studies.

**Table 3:** Comparison with other studies SD: standard deviation, PR: partial response, SD: stable disease, PR: partial response, PFS: progression-free survival, OS: overall survival

	SHARP study [2]	REFLECT study [10]	Asia-Pacific study [3]	IMbrave150 [12]	Ostwal V et al. [13]	Our study
Study period	2005-2006	2013-2015	2005-2017	2018-2019	2016	2017-2022
Number of patients taking sorafenib	299	476	150	165	39	80
Age in years	64.9 (11.2) Mean (SD)	62 (22-88) Median (range)	51 (23-86) Median (range)	66 (59-71) Median (range)	58 (20-75) Median (range)	62 (17-79) Median (range)
Barcelona stage B	54 (18%)	92 (19%)		26 (16%)		19 (23.7%)
Barcelona stage C	244 (82%)	384 (81%)	145 (95.3%)	133 (81%)	39 (100%)	61 (76.2%)
Child-Pugh A	284 (85%)	471 (99%)	146 (97.3%)	165 (100%)	35 (89.7%)	55 (68.8%)
Child-Pugh B	14 (5%)	5 (1%)	4 (2.7%)		4 (10.3%)	25 (31.2%)
PR	2%	6%	3.3%	11.9%		7.5%
SD	71%	53%	54%	43.4%		48.7%
Discontinuation rate	38%	7%	19.5%	10.3%	41%	7.5%
Median PFS (months)	4.1	3.7	2.8	4.3	4.2 (EFS)	3.68
Median OS (months)	10.7	12.3	6.5			5.26

Clinical trial dosage schedules are often created using the maximum tolerable dose (MTD), which was first used with conventional cytotoxic chemotherapy. Patients often require time to recover from dose-limiting toxicities, this may not be possible in malignancies like HCC. With more targeted treatments becoming clinically available, there has been a growing interest in the concepts of minimal effective dose (MED), optimal biological dosage, and biologically efficacious dosing. According to a case series from Cleveland, Ohio, three distinct HCC patients experienced clinical responses that were longer than those documented in their respective phase 3 clinical trials on 1/8th the target dose for sorafenib, 1/4th the target dose for regorafenib, and 1/6th the target dose for cabozantinib, respectively [2,14-16]. In the post hoc analysis of the SOFIA (SOraFenib Italian Assessment) study, patients who were administered a half-dose of sorafenib for over 70% of the treatment duration exhibited improved survival rates compared to those who received either a full dose or a half dose of sorafenib for less than 70% of the treatment period, with survival times of 21.6 months versus 9.6 months, respectively [17]. Similar to our study, they also advocate for maintaining compliance and the continuation of sorafenib treatment, even at reduced dosages. Results from the Canadian Multicenter database show that a reduced dose of sorafenib compared to a full dose may not compromise survival (9.4 vs 8.9 months, p-0.15) [5].

Our study has several strengths. These patients were not part of a clinical trial, making them a more accurate representation of the real-world population affected by HCC. Nearly one-third of our study patients belonged to Child-Pugh B status. No studies have studied the effectiveness of low-dose tyrosine kinase in the Asian population. The main limitation of our study is its retrospective nature, and that, very few patients received subsequent treatment after sorafenib.

## Conclusions

In comparison to other published landmark studies, our study demonstrates that reduced dose sorafenib in advanced hepatocellular carcinoma has a similar response rate and progression-free survival with lesser toxicity. In the real world, a reduced dose of sorafenib is nevertheless effective when tolerance and cost are concerns. Additionally, since a third of the study cohort has Child-Pugh B, a reduced dose of sorafenib may be a choice for these patients.
